# Vick’s Floral Guide

**Published:** 1875-01

**Authors:** 


*Vick's Floral Guide*

For 1875 is a beauty. We would advise our readers to send 25 cents to James Vick, of Rochester, New York, and get it for the year. Vick is the most reliable seedsman within our knowledge. He always gives satisfaction.

